# miR-7 Knockdown by Peptide Nucleic Acids in the Ascidian *Ciona intestinalis*

**DOI:** 10.3390/ijms20205127

**Published:** 2019-10-16

**Authors:** Silvia Mercurio, Silvia Cauteruccio, Raoul Manenti, Simona Candiani, Giorgio Scarì, Emanuela Licandro, Roberta Pennati

**Affiliations:** 1 Department of Environmental Science and Policy, Università degli Studi di Milano, 20133 Milano, Italy; sil.mercurio@gmail.com (S.M.); raoul.manenti@unimi.it (R.M.); roberta.pennati@unimi.it (R.P.); 2 Department of Chemistry, Università degli Studi di Milano, 20133 Milano, Italy; emanuela.licandro@unimi.it; 3 Department of Earth Science, Environment and Life, Università degli Studi di Genova, 16126 Genova, Italy; 4 Department of Biosciences, Università degli Studi di Milano, 20133 Milano, Italy; nkiller@unimi.it

**Keywords:** microRNA, *hnRNP K*, PNA, tunicates, LNA probe

## Abstract

Peptide Nucleic Acids (PNAs) are synthetic mimics of natural oligonucleotides, which bind complementary DNA/RNA strands with high sequence specificity. They display numerous advantages, but in vivo applications are still rare. One of the main drawbacks of PNAs application is the poor cellular uptake that could be overcome by using experimental models, in which microinjection techniques allow direct delivery of molecules into eggs. Thus, in this communication, we investigated PNAs efficiency in miR-7 downregulation and compared its effects with those obtained with the commercially available antisense molecule, Antagomir (Dharmacon) in the ascidian *Ciona intestinalis*. Ascidians are marine invertebrates closely related to vertebrates, in which PNA techniques have not been applied yet. Our results suggested that anti-miR-7 PNAs were able to reach their specific targets in the developing ascidian embryos with high efficiency, as the same effects were obtained with both PNA and Antagomir. To the best of our knowledge, this is the first evidence that unmodified PNAs can be applied in in vivo knockdown strategies when directly injected into eggs.

## 1. Introduction

**Figure 1:**
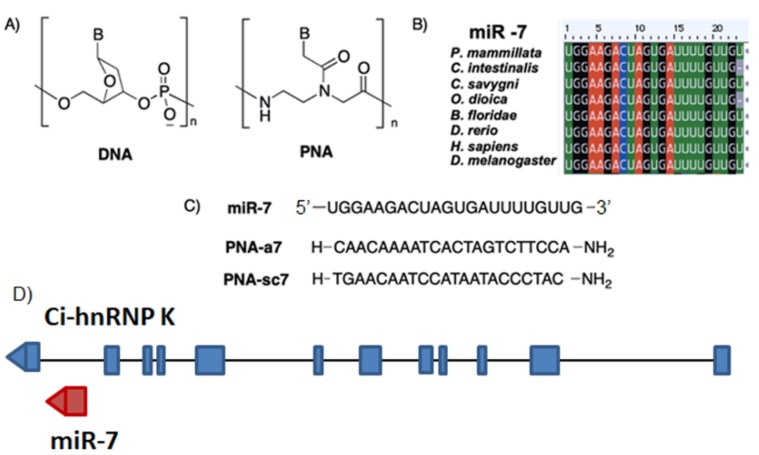
(**A**) DNA and Peptide Nucleic Acids (PNA) backbone; (**B**) multi-alignment of mature miR-7 sequences in different species: *Phallusia mammillata*, *Ciona intestinalis*, *Ciona savignyi*, *Oikopleura dioica*, *Branchiostoma floridae*, *Danio rerio*, *Homo sapiens*, *Drosophila melanogaster*; (**C**) miR-7 and PNAs sequences used in this study; (**D**) schematic representation of miR-7 genomic position inside the last Ci-hnRNP K intron (blue rectangles and interconnecting lines represent exons and introns, respectively; the red graph corresponds to the miR-7 sequence).

Peptide Nucleic Acids (PNAs) are artificial nucleic acids mimics [1], extensively used for the regulation of gene expression in cellular and molecular systems [2]. In PNAs, the neutral pseudo-peptide backbone, based on *N*-(2-aminoethyl)glycine units (Figure 1A), replaces the negatively charged sugar-phosphate chain of nucleic acids. PNAs can recognize and bind to DNA or RNA sequences according to regular Watson–Crick base pairing rules [3]. Unlike DNA or RNA, PNAs are chemically stable across a wide range of temperatures and pHs, and they are resistant to enzymatic degradation since they are not easily recognized by nucleases or proteases [4]. Moreover, one of the most remarkable properties of PNA is the excellent thermal stability of PNA/DNA and PNA/RNA duplexes, in comparison with DNA/DNA or DNA/RNA duplexes [5]. Indeed, the lack of charge repulsion between the neutral PNA strand and the DNA or RNA strand provides extremely stable complexes. For example, Shakeel et al. reported that the melting temperature (*T_m_*) values for a 15-mer PNA/DNA or PNA/RNA duplex are generally 20 °C higher than the natural nucleic acid duplexes [6]. All these properties make PNAs excellent candidates for in vivo antisense and antigene therapies, targeting oncogenes, viruses, and bacteria [7]. Indeed, studies on the use of PNAs in knockdown technologies are accumulating, demonstrating PNAs potential for future therapeutic purposes, as well as for basic research. However, in vivo PNAs applications are still rare [8,9] due to some drawbacks, such as poor cellular uptake and low solubility in aqueous media [10], which can be improved either by conjugation with carrier molecules or by chemical modifications [11].

PNAs poor cellular uptake could be also overcome by using model organisms, such as ascidians, in which molecules can be directly injected in the target tissue/cell. Ascidians are marine invertebrates, closely related to vertebrates [12]. They develop through a swimming larva that shows the basic chordate features, comprising a notochord, which runs along the tail, and a dorsal tubular central nervous system (CNS) [13,14]. Particularly, the ascidian *Ciona intestinalis* is amenable to embryological manipulations. A variety of molecular tools were developed to perturb gene activity during its development, including microinjections of antisense molecules directly into unfertilized eggs [15,16].

To verify PNAs efficiency in gene downregulation during ascidian development, we chose one of the most evolutionarily conserved microRNAs (miRNAs), miR-7 [17], as PNAs target. miRNAs are a class of non-coding RNAs that regulate gene expression at post-transcriptional level. They are found in all animal lineages, where they modulate multiple biological processes [18,19]. A single miRNA has the potential to target a broad spectrum of mRNAs, possessing great regulatory potential [20]. However, in many cases, knockout of individual miRNA does not lead to critical effects, as the same pathway is often controlled by many of these molecules that collectively affect the pathway by exerting fine-tune functions and ensuring the correct progression of cellular and developmental programs [21].

In mammals, miR-7 is expressed predominantly in the pancreas, neural tissues and pituitary [22,23]. miR-7 is highly expressed in neurons with sensory or neurosecretory functions in fish and animals distantly related from vertebrates, such as annelids [24]. miR-7 expression in photoreceptors is similarly conserved, being reported in rodents [25], amphioxus [26], and even *Drosophila* [27]. A gene encoding for miR-7 is also present in the genome of the ascidian *C. intestinalis* (www.mirbase.org), but its expression has not been described yet.

Thus, in this communication, we aim to characterize miR-7 expression profile in the ascidian *C. intestinalis*, and then test PNAs in vivo knockdown efficiency in this species, comparing it with the commercial antisense molecule, Antagomirs (Dharmacon, USA) [28].

## 2. Results

### 2.1. miR-7 in Ciona intestinalis

Comparing miR-7 mature sequences in different animal models, we observed that miR-7 is highly conserved also in basal chordates: *C. intestinalis* miR-7 differs from that of *Homo sapiens*, only by the deletion of the terminal uracil. This feature is shared with another tunicate species, *Oikopleura dioica*, while in the available transcriptomes of two other ascidians, miR-7 mature sequences are completely conserved (Figure 1B).

In *C. intestinalis* genome, the miR-7 gene resides within the last intron of the heterogeneous nuclear ribonucleoprotein K (hnRNP K) gene, oriented in the same direction as the Ci-hnRNP K transcription unit (Figure 1D).

### 2.2. Genes Expression Profile

**Figure 2:**
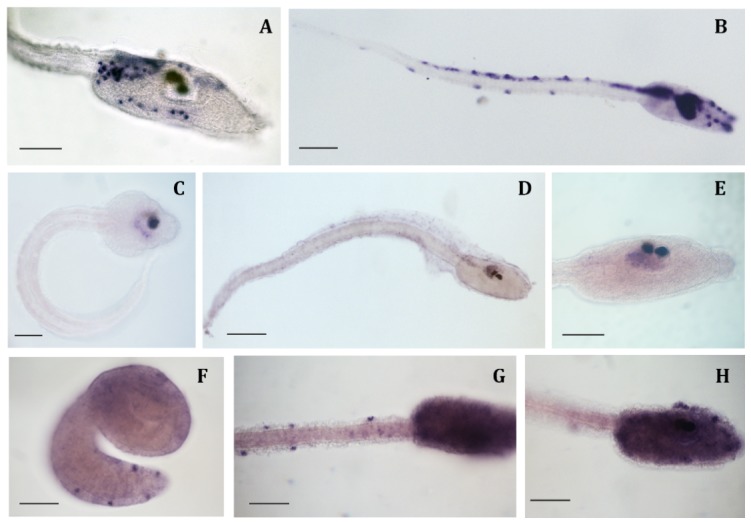
Whole mount in situ hybridization of *C. intestinalis* embryos. (**A**) Unspecific stain in mesenchymal cells of larva trunk, obtained when hybridization with LNA probes was performed overnight; (**B**) miR-124 expression in larval central and peripheral nervous system; (**C**) miR-7 expression at late tailbud stage: signal is clearly visible in the posterior ventral part of the sensory vesicle; (**D**,**E**) miR-7 expression at larva stage: the signal persists in the posterior ventral region and faintly extends in the neural ganglion; (**F**) hnRNP K expression at mid tailbud stage: signal appears more intense in the epidermal sensory neurons; (**G**,**H**) hnRNP K expression at larva stage: signal persists in epidermal sensory neurons and extends all over the trunk. (**A**,**E**–**H**) Scale bar = 60 µm; (**B**–**D**) Scale bar = 100 µm.

To determine the expression pattern of miR-7 mature transcripts during *C. intestinalis* development, standard in situ hybridization protocol [29] was ineffective. When hybridization with DIG-labeled Locked Nucleic Acid (LNA; Exiqon, Vedbaek, Denmark) probes were carried out overnight, unspecific stains were always detected in mesenchymal cells of late tailbud and larva trunk (Figure 2A). Extending the hybridization step to five days and increasing the hybridization temperature (5 °C more than the recommended temperature) was found to be optimal for miRNA detection with LNA probes, as confirmed by miR-124 results (Figure 2B). Performing this modified protocol, we found that miR-124 mature transcripts were abundantly present in all of the nervous system of *C. intestinalis* larva, as previously reported by Zeller and co-workers [30].

Using this protocol, we found that miR-7 expression started in the central nervous system at the late tailbud stage, but only in the ventral posterior part of the sensory vesicle (Figure 2C). At larval stage, the signal persisted in this region and faintly extended in the neural ganglion (Figure 2D,E).

hnRNP K, the gene hosting miR-7, was ubiquitously expressed at early developmental stages; but from mid tailbud stage, the signal was more intense in the epidermal sensory neurons, i.e., ascidian peripheral nervous system, of both trunk and tail (Figure 2F). This expression persisted at the late tailbud and larval stages, and a strong signal was also detectable all over the trunk (Figure 2G,H).

### 2.3. miR-7 Downregulation by PNAs

To evaluate PNAs effectiveness in miRNA knockdown, we designed a 22-mer PNA complementary to *C. intestinalis* miR-7 (PNA-a7, Figure 1C) and a PNA scrambled sequence with the same base composition of PNA-a7 (PNA-sc7, Figure 1C). Then, PNA-a7 and PNA-sc7 were microinjected in *C. intestinalis* eggs before in vitro fertilization. Moreover, we performed microinjections employing the commercial AntagomiR (AmiR-7), commonly used in miRNAs knockdown studies [28], and we compared the effects with those obtained with PNAs.

Preliminary trials revealed that the highest non-lethal concentrations were 0.7 mM for PNAs (PNA-a7 and PNA-sc7) and 0.3 mM for AmiR-7. Based on these results, PNAs solution seemed less toxic than AmiR-7, as embryos injected with concentrations higher than 0.3 mM AmiR-7 died before completing embryogenesis. All the following analyses were performed on embryos injected with 0.7 mM PNAs or 0.3 mM AmiR-7.

The developing rates of controls (injected with only the vital dye, Fast Green) and embryos injected with PNA-sc7, PNA-a7 or AmiR-7 were comparable, ranging from 69% (PNA-a7) to 72% (PNA-sc7) and no difference in sample morphology was recorded.

The specificity of PNA-a7 as well as AmiR-7 for miR-7 in *C. intestinalis* was previously checked by blast search in its genome by using cin-miR-7 (MIMAT0006091) as query. No identity with other microRNAs were found. Few mRNAs (for example: NLRC5-like or FAM192A-like mRNAs) having some sequence identity with miR-7 were obtained but the query coverage was lower, as there was some similarity but not for the entire sequence of miR-7.

**Figure 3:**
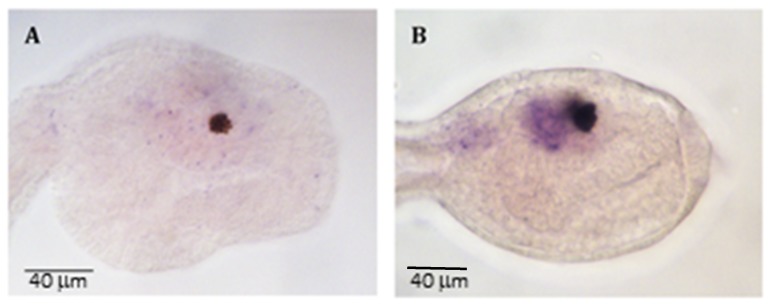
*C. intestinalis* miR-7 expression at the late tailbud stage in embryos injected with (**A**) PNA-a7 and (**B**) PNA-sc7. Scale bar = 40 µm.

To verify miRNA downregulation by PNAs, we first evaluated miR-7 expression by in situ hybridization. Results revealed that miR-7 expression was drastically reduced in embryos injected with PNA-a7 (Figure 3A), while miR-7 was normally expressed in embryos injected with PNA-sc7 (Figure 3B).

**Figure 4:**
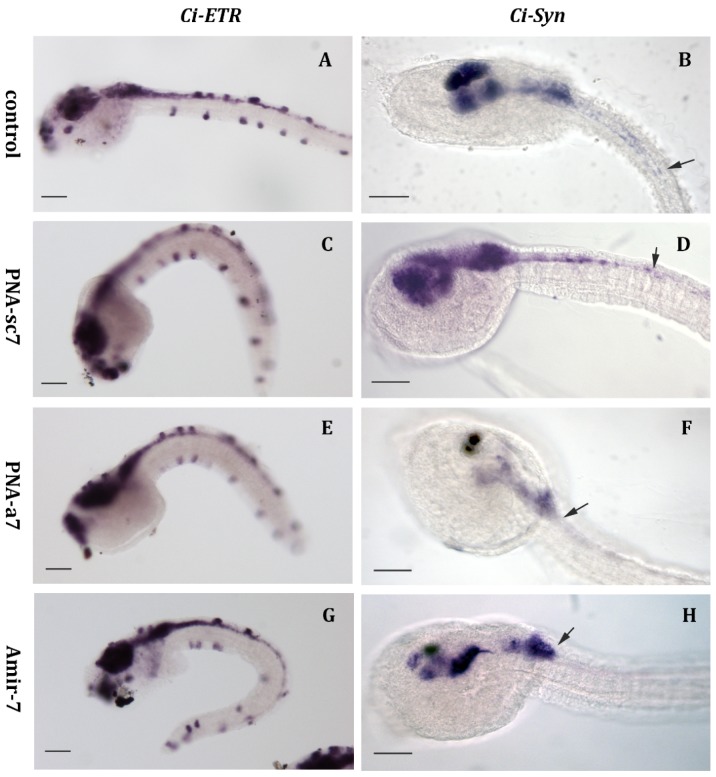
Whole mount in situ hybridization of *C. intestinalis* control embryos and embryos injected with PNA-sc7, PNA-a7, and AntagomiR (AmiR-7). (**A**,**C**,**E**,**G**) Ci-ETR expression in central and peripheral nervous system of late tailbud embryos; (**B**,**D**,**F**,**H**) Ci-Syn expression at late tailbud stage: in control and PNA-sc7 injected embryos, signal is detectable in most of the sensory vesicle, in the motor ganglion and along the posterior neural tube. In embryos injected with PNA-a7 and AmiR-7, transcripts are present only in a subpopulation of neurons in the sensory vesicle and in the motor ganglion, while no signal is recorded in posterior neural tube. Arrows indicate the posterior limit of the signal. (**A**,**C**,**E**,**G**) Scale bar = 15 µm. (**B**,**D**,**F**,**H**) Scale bar = 10 µm.

Then, we checked the expression of some pan-neural genes: Ci-ETR [31] and Ci-Syn [32] in all the injected embryos. Ci-ETR expression was normal as its signal was observed throughout the central nervous system and in the epidermal sensory neurons of all injected samples (Figure 4A,C,E,G). Nevertheless, the expression of Ci-Syn was reduced in embryos injected with PNA-a7 (87%, *n* = 28) and AmiR-7 (91%, *n* = 32), compared to PNA-sc7 (*n* = 41). In control embryos and in embryos injected with PNA-sc7, the hybridization signal occurred in most of the sensory vesicle, in the motor ganglion, and extended into the posterior neural tube (Figure 4B,D). In embryos injected with PNA-a7, Ci-Syn transcripts were detected only in a subpopulation of neurons in the sensory vesicle and in the motor ganglion, and not detected in the posterior neural tube (Figure 4F). The same expression pattern was present in embryos injected with AmiR-7 (Figure 4H).

## 3. Discussion

PNAs are synthetic mimics of natural oligonucleotides, which bind complementary DNA/RNA strands with high sequence specificity [3]. In comparison with DNA/DNA or DNA/RNA duplexes, PNA/DNA and PNA/RNA duplexes show excellent thermal stability due to the neutral PNA backbone that lacks a repulsion charge when binding with DNA or RNA, providing more stable complexes [5]. Although they display numerous advantages, in vivo applications are still rare due to their poor cellular uptake. The latter, however, could be overcome by using experimental models, such as the ascidian *C. intestinalis*. In this model organism, microinjections allow direct delivery of antisense molecules into eggs, perturbing gene activity during embryonic development [16]. To verify this hypothesis, we chose miR-7 as PNAs target. miRNAs are potent endogenous regulators of gene expression with fundamental roles in development [21].

In *C. intestinalis*, hundreds of miRNAs have been identified (www.mirbase.org) but expression data are reported only for miR-124 [30]. In fact, characterization of miRNAs expression is extremely challenging due to their tiny size and low level of expression. When performing in situ hybridization, the possibility to get nonspecific signals is rather high. Thus, we first optimized the hybridization protocol with DIG-labeled LNA (Exiqon) probes to obtain specific staining even when miRNA levels are particularly low, as in miR-7′s case. In particular, modifications in hybridization temperature and incubation time were found to highly improve miRNAs detection.

Employing this protocol, we described for the first time miR-7 expression during ascidian development. In *C. intestinalis*, miR-7 mature transcripts were detected at late tailbud and larva stages in the central nervous system, particularly, in the ventral posterior part of the sensory vesicle (Figure 2C–E). miR-7 neural expression has been reported in different animal models, specifically in photoreceptors and/or neurosecretory tissues [24,26,33]. miR-7 is considered part of the evolutionary conserved fingerprint of neurosecretory cells [24]. In *C. intestinalis*, the ventral region of the larval sensory vesicle has been proposed to be homologous to vertebrate hypothalamus and retinal amacrine cells [34,35,36]. Thus, miR-7 expression also appears highly conserved in ascidians. Its expression domain, together with that already reported for Ci-Rx [37], Ci-Nk2, and Ci-Otp, [35] further supports the homology of this region to vertebrate hypothalamus [24,33,35]. Moreover, miR-7 was reported to be expressed in mammalian retina; and in ascidians, miR-7 was identified in the photoreceptive neuroepithelium [36], further indicating the striking evolutionary conservation of this miRNA among chordates.

In addition, miR-7 genomic position seems extremely conserved. In C. *intestinalis* genome, miR-7 was found within the last intron of the heterogeneous nuclear ribonucleoprotein K (hnRNP K) gene, similarly to those already reported for hsa-miR-7-1 in *Homo sapiens* [38] and in *Drosophila* [39].

hnRNP K is referred as a ubiquitously expressed gene involved in different aspects of RNA functions: transcription, editing, processing, and translation [40]. During vertebrate embryogenesis hnRNP K is uniformly expressed in all blastomeres; and only at later stages, transcripts appear more concentrated in specific tissues, such as central nervous system and mesodermal derivatives [41]. In *C. intestinalis*, Ci-hnRNP K displays a similar expression during early developmental stages; while at the late tailbud and larva stages, transcripts accumulate in the epidermal sensory neurons (Figure 2F–H), i.e., ascidian peripheral nervous system. Like in vertebrates, Ci-hnRNP K expression does not overlap with that of its host miR-7. Since genes are oriented in the same direction (Figure 1D), post-transcriptional regulation of miR-7 biogenesis is likely to occur, as demonstrated in both mice and humans [38].

Then, we designed a 22-mer PNA complementary to *C. intestinalis* miR-7 (PNA-a7, Figure 1C) as well as a PNA scrambled sequence (PNA-sc7, Figure 1C), to verify the specificity of the interaction between PNA-a7 and miR-7. Our hybridization analysis confirmed that PNA-a7 efficiently downregulates miR-7 and that PNAs interaction occurs in a sequence-specific manner, as samples injected with PNA-sc7 never showed a similar signal reduction (Figure 3). To further verify PNAs effectiveness, we compared the effects induced by miR-7 downregulation by injecting the commercial AntagomiR molecules, commonly used in miRNAs knockdown studies (AmiR-7) [28], and our PNAs. We checked the expression of two pan-neural genes, Ci-ETR [31] and Ci-Syn [32]. Ci-ETR signal was not affected by neither the PNAs nor AmiR-7 injection, suggesting that perturbation of miR-7 expression does not affect nervous system differentiation. On the contrary, Ci-Syn signal was reduced in the posterior neural tube of embryos injected with PNA-a7 and AmiR-7 (Figure 4F,H), but not in those injected with PNA-sc7. Synapsins are neuronal phosphoproteins that constitute a small family of synaptic molecules, specifically associated with synaptic vesicles. They exert a key role in neurite outgrowth and synapse formation [32]. In human neural embryonic stem cells, miR-7 overexpression during their neuronal differentiation increased synapsin expression. Synapsin mRNA is not a direct target of miR-7 but miR-7 levels are positively correlated with synapsin expression, suggesting that this miRNA could act upstream of the synapsin pathway, and play an important role in synaptic development [39]. Similarly, in *C. intestinalis* embryos, Ci-Syn expression decreased after miR-7 knockdown, indicating that miR-7 has a functional role in synaptic plasticity and neurite elongation in ascidians.

Comparable results were obtained with both PNA-a7 and AmiR-7, confirming the reliability of our results and the specificity of PNAs. These are in agreement with different in vitro research, which demonstrated PNAs specificity for their complementary miRNAs [42]. In vivo experiments using modified PNAs have been performed in mouse: miR-155 inhibition by PNAs was demonstrated to be sequence specific, not affecting levels of unrelated miRNAs and mainly recapitulating the effects of genetic deletion of miR-155 [8].

## 4. Materials and Methods

### 4.1. Animals and Embryos Culture

Adult *Ciona intestinalis* were collected along the coasts of Roscoff (France) by the fishing service of the Station Biologique de Roscoff. Animals were maintained in aquaria filled with artificial sea water (Instant Ocean; salinity ~32‰) and provided with a circulation system, as well as mechanical, chemical, and biological filters. Constant light conditions were preferred to promote gamete production [29]. For each experiment, gametes from three adults were obtained surgically from the gonoducts and in vitro cross-fertilization was performed. Embryos were reared in petri dishes in filtered artificial sea water buffered with 1 M HEPES (ASWH; pH 8.0) at 18 ± 1 °C up to the stages of interest (gastrula stage; neurula stage; initial, early, mid, and late tailbud stages; larva stage). Then, they were dechorionated in ASWH containing 1% sodium thioglycolate and 0.05% protease; fixed in 4% paraformaldehyde, 0.5 M NaCl, and 0.1 M 3-(*N*-morpholino)propanesulfonic acid (pH 7.5) for 90 min; dehydrated in ethanol series (30%, 50%, and 70%); and stored at −20 °C.

### 4.2. Reagents

Antagomirs are chemically modified, cholesterol-conjugated single-stranded RNA analogues complementary to the mature miRNA sequences, commonly applied in miRNAs knockdown research [28]. Based on *C. intestinalis* sequence, a specific anti-miR-7 antagomir (AmiR-7: 5′- C_S_A_S_ACAAAAUCACUAGUCUU_S_C_S_C_S_A_S_-Chol-3′) was designed and synthetized by Dharmacon (USA).

### 4.3. PNAs Synthesis and Characterization

Two PNA oligomers were designed: PNA-a7 complementary to mature miR-7; PNA-sc7 with a scrambled sequence, but the same base composition of PNA-a7 as control (Figure 1C). They were synthesized by automated solid-phase synthesis using Boc/Z chemistry by means of the automated synthesizer, Applied Biosystems 433A Peptide Synthesizer (Monza, Milan, Italy), equipped with Synthassist 2.0 software. The commercially available Boc/Z-protected PNA monomers were purchased from ASM Research Chemicals GmbH (Hannover, Germany). The MBHA resin was purchased from VWR International, and it was loaded manually to 0.2 mmol/g with Boc/Z-adenine PNA monomer for PNA-a7, and with Boc/Z-cytosine PNA monomer for PNA-sc7 [43]. The PNA purification was performed using reverse phase high pressure liquid chromatography (RP-HPLC) with an Agilent 1200 Series system (Cernusco sul Naviglio, Milan, Italy), equipped with DAD analyzer (UV detection at 260 and 280 nm, Cernusco sul Naviglio). The purity of PNA-a7 and PNA-sc7 was checked by RP-HPLC analyses, and their identity was confirmed by electrospray-ionisation quadrupole time-of-flight mass spectrometry (ESI-Q-TOF MS) mass analysis (Q-Tof Micro, Waters). PNA-a7, calculated MW: 5875.4; ESI-MS: m/z found (calculated): 1470.1 (1469.9) [MH_4_^4+^], 1176.2 (1176.1) [MH_5_^5+^], 980.4 (980.2) [MH_6_^6+^], 840.5 (840.3) [MH_7_^7+^], 735.5 (735.4) [MH_8_^8+^], 653.9 (653.8) [MH_9_^9+^]. PNA-sc7, calculated MW: 5875.4; ESI-MS: m/z found (calculated): 1470.0 (1469.9) [MH_4_^4+^], 1176.2 (1176.1) [MH_5_^5+^], 980.3 (980.2) [MH_6_^6+^], 840.4 (840.3) [MH_7_^7+^], 735.5 (735.4) [MH_8_^8+^], 653.9 (653.8) [MH_9_^9+^].

The melting temperature (*T_m_*) of PNA-a7/DNA duplex was calculated according to the linear model for the melting temperature prediction of PNA/DNA duplexes [44]. In particular, taking into account the following formula:

*T_m_*_, pred_ = *c*_0_ + *c*_1_ × *T_m_*_, nnDNA_ + *c*_2_ × *f*_pyr_ + *c*_3_ × *length* (1)

in which *T_m_*_, nnDNA_ is the melting temperature as calculated using the nearest neighbor model for the corresponding DNA/DNA duplex, applying Δ*H*^0^ and Δ*S*^0^ values as described by SantaLucia et al. [45], *f*_pyr_ denotes the fractional pyrimidine content, *length* is the PNA sequence length in bases, and the constants were determined to be *c*_0_ = 20.79, *c*_1_ = 0.83, *c*_2_ = –26.13, *c*_3_ = 0.44. The calculated *T_m_*_, pred_ of PNA-a7 was found to be 65.2 °C.

### 4.4. Microinjections

For microinjections, only batches in which 90% or more of the embryos developed normally were used. Concentrations of injected solutions were determined by preliminary experiments. We tested the following concentrations: 0.3, 0.5, and 0.7 mM of PNAs (PNA-a7 and PNA-sc7); and 0.3 and 0.5 mM of AmiR-7. For each molecule, the maximum non-lethal concentration was chosen. Dechorionated eggs were microinjected with a solution of 0.7 mM PNAs (PNA-a7 or PNA-sc7) in distilled water or 0.3 mM AmiR-7 plus 5 μg/μL Fast Green as vital dye, as previously described [15]. Embryos were reared at 18 ± 1 °C until they reached late tailbud stage [46].

### 4.5. Whole Mount In Situ Hybridization

To describe gene expression during development and evaluate microinjection effects, a standard protocol for whole mount in situ hybridization (WISH) was employed [29] with some modifications. Dechorionated embryos and larvae were permeabilized with 2 μg/mL proteinase K in PBS + 0.1% Tween20 for 5 min at 37 °C. To detect miR-7 mature transcripts (MIMAT0003552), a hybridization step was carried out with a DIG-labeled Locked Nucleic Acid (LNA) probe (cin-miR-7-5p: 5′-UGGAAGACUAGUGAUUUUGUUG; RNA *T_m_* = 76 °C) for 5 days at 50 °C. The specificity of the miR-7 signal was confirmed by results obtained using the LNA probe against *C. intestinalis* miR-124, whose expression pattern is well known [30]. The riboprobe specific for hnRNP K was obtained from a GC27a23 plasmid contained in the *C. intestinalis* gene collection release I [47]. DIG-labelled riboprobes were transcribed with Sp6 (antisense) and T7 (sense) RNA polymerase, using a DIG RNA labelling kit (Roche, Monza, Italy). Microinjection effects were explored employing riboprobes against the pan-neural marker Ci-ETR [31] and the gene Ci-Syn, encoding for synapsin, a protein specifically associated with synaptic vesicles [32]. For each probe, at least 40 injected and control embryos were analyzed.

## 5. Conclusions

Overall, our results demonstrate the in vivo biological activity of PNA oligomers directed against miR-7 in *C. intestinalis* embryos. This animal model allowed direct injection of the anti-miR PNA in eggs, overcoming the typical drawbacks associated with the PNAs poor cellular uptake [48]. One still open problem in antisense approaches is the way of delivering antisense molecules to their target cells in a complex organism. Our results suggest that PNA-a7 is able to reach its specific target in the developing ascidian embryos with high efficiency, as underlined by the lack of effects induced by the scrambled sequence PNA-sc7. To the best of our knowledge, this is the first evidence that unmodified PNA can be successfully used in knockdown strategies in a multicellular organism.

Moreover, our results could be the basis for future quantitative analyses investigating in detail the effect of PNAs.

## Abbreviations

ASWH	Artificial Sea Water with HEPES
AmiR-7	AntagomiR anti-miR-7
Boc	*tert*-Butyloxycarbonyl
ESI-Q-TOF MS	Electrospray-ionisation quadrupole time-of-flight mass spectrometry
LNA	Locked Nucleic Acid
MBHA	4-Methylbenzhydrylamine hydrochloride
PNA	Peptide Nucleic Acid
PNA-a7	Peptide Nucleic Acid anti-miR-7
PNA-sc7	Peptide Nucleic Acid scrambled sequence
RP-HPLC	Reverse phase-high pressure liquid chromatography
Z	Benzyloxycarbonyl
